# Protocol for volume correlative light X-ray and electron microscopy of endothelial cells in mouse tissue

**DOI:** 10.1016/j.xpro.2024.103257

**Published:** 2024-09-01

**Authors:** Natalia Reglero-Real, Lorena Pérez-Gutiérrez, Rebeca S. Saleeb, Sussan Nourshargh, Lucy Collinson, Azumi Yoshimura

**Affiliations:** 1 Centre for Microvascular Research, William Harvey Research Institute, Barts and the London School of Medicine and Dentistry, Queen Mary University of London, EC1M 6BQ London, UK; 2 Electron Microscopy Science Technology Platform, The Francis Crick Institute, NW1 1AT London, UK; 3 Departamento e Instituto de Biología Molecular (IUBM, UAM), Centro de Biología Molecular Severo Ochoa, Universidad Autónoma de Madrid, UAM-CSIC, Campus de Cantoblanco, 28049 Madrid, Spain; 4 Cancer Immunology and Immunotherapy Lab, Center for Cooperative Research in Biosciences (CIC BioGUNE), Basque Research and Technology Alliance (BRTA), Derio, 48160 Bizkaia, Spain; 5 Institute of Regeneration and Repair, University of Edinburgh, EH16 4UU Edinburgh, UK; 6 Graduate School of Medicine, Yamaguchi University, Ube 755-8505, Japan

**Keywords:** Cell Biology, Immunology, Microscopy

## Abstract

Correlative light and electron microscopy (CLEM) greatly facilitate capturing the ultrastructure of spatially and/or temporally rare events. Here, we present a protocol for targeting regions of interests (ROIs) in tissue endothelial cells (ECs) using X-ray micro-computed tomography (μCT). We describe steps for ROI targeting guided by vasculature patterns and positions of EC nuclei visualized by light and X-ray microscopy. The protocol is applicable to thin or translucent tissues that contain defined landmarks visible in both light and X-ray microscopy.

For complete details on the use and execution of this protocol, please refer to Reglero-Real et al.^1^

## Highlights

• Instructions for *in vivo* fluorescence labeling of tissue ECs
• Procedures for confocal microscopy to achieve successful ROI targeting
• Steps for sequential trimming and X-ray μCT to obtain the final block for SBF-SEM
• Guide for data correlation and ROI targeting using open-source programs

## Abstract (editor highlights)

Publisher’s note: Undertaking any experimental protocol requires adherence to local institutional guidelines for laboratory safety and ethics.

## Graphical abstract

**Figure:**
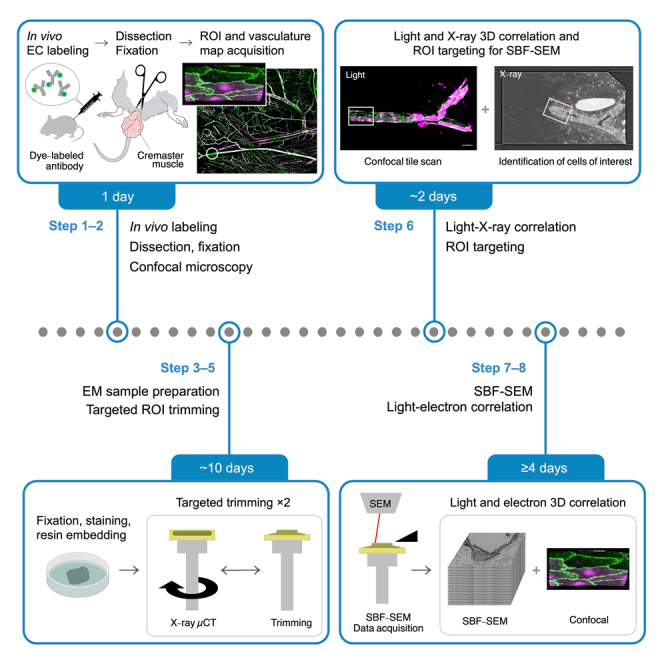

## Before you begin

For accurate 3D correlation of light microscopy and X-ray μCT data, the EC nuclei must be labeled with fluorescent probes prior to imaging. In the example included to illustrate this protocol, a mouse model expressing the red fluorescent protein variant tdTomato in ECs has been used. This protein localizes to the cell nucleus and cytoplasm. However, as long as they label EC nuclear morphology, other fluorescent reporters can be equally used.

The genetically modified mice used in the protocol below exhibit Cre-recombinase expression under the EC specific *Cdh5* promoter, the Rosa-26-tdTomato Cre reporter as mentioned above, and floxed alleles for the autophagy related protein 5 (*Atg5*) (*Cdh5-Cre*; *Atg5*^*fl/fl*^; *Rosa26*^*tdTomato/tdTomato*^; hereafter referred to as *Atg5Δ*^*EC*^ mice). Since tdTomato expression and *Atg5* deletion is achieved following *Cdh5-Cre* mediated recombination, this mouse line exhibited genetically mosaic populations of ECs comprised of tdTomato- (WT) ECs and neighboring tdTomato+ (*Atg5*^*−/−*^ or autophagy deficient) ECs.^1^

### Institutional permissions

All animal procedures were performed using 8-12-week-old mice on a C57BL/6 background (age and sex matched groups) in accordance with the institutional Animal Welfare Ethical Review Body (AWERB) and UK Home Office guidelines.

## Key resources table

**Table:** 

REAGENT or RESOURCE	SOURCE	IDENTIFIER
**Antibodies**		

Anti-mouse PECAM-1 (clone 390)	Thermo Fisher Scientific	16-0311-85; RRID: AB_468933

**Chemicals, peptides, and recombinant proteins**		

Dulbecco phosphate-buffered saline	Sigma-Aldrich	D8537
Disodium hydrogen phosphate, anhydrous	VWR Chemicals	102494C
Sodium dihydrogen phosphate dihydrate	VWR Chemicals	28015.261
36% w/v formaldehyde EM	TAAB	F003
25% w/v glutaraldehyde EM	TAAB	G004
Osmium tetroxide 4% aqueous solution	TAAB	O012
Potassium ferricyanide (K_3_Fe(CN)_6_)	Sigma-Aldrich	P-9387
Thiocarbohydrazide	Sigma-Aldrich	88535
Uranyl acetate	Agar Scientific	AGR1260A
Lead nitrate	Sigma-Aldrich	228621
Aspartic acid	Sigma-Aldrich	A4534
Potassium hydroxide	Sigma-Aldrich	60377
Ethanol (absolute, 99.8+%)	Fisher Chemical	E/0650DF/17
Propylene oxide	TAAB	P021
Durcupan ACM, single component A, M epoxy resin	Sigma-Aldrich	44611
Durcupan ACM, single component B, hardener 964	Sigma-Aldrich	44612
Durcupan ACM, single component C, accelerator 960 (DY 060)	Sigma-Aldrich	44613
Durcupan ACM, single component D	Sigma-Aldrich	44614

**Critical commercial assays**		

Alexa Fluor 488 antibody labeling kit	Thermo Fisher Scientific	A20181

**Deposited data**		

Confocal microscopy data	BioStudies	S-BSST1339
X-ray μCT and SBF-SEM data	EMPIAR	EMPIAR-11958

**Experimental models: Organisms/strains**		

*Mus musculus C57BL/6* *Cdh5-cre; Atg5*^*fl/fl*^*; Rosa26*^*tdTomato/tdTomato*^ 2- to 4-week-old males	Reglero-Real et al.^1^	https://doi.org/10.1016/j.immuni.2021.07.012

**Software and algorithms**		

3dmod program of the IMOD software package	Kremer et al.^2^	https://bio3d.colorado.edu/imod/
Photoshop	Adobe	
Fiji (imageJ)	Schindelin et al.^3^	https://imagej.net/Fiji
BigWarp	Bogovic et al.^4^	https://imagej.net/plugins/bigwarp
Register Virtual Stack Slices	https://biii.eu/register-virtual-stack-slices-imagej	https://imagej.net/plugins/register-virtual-stack-slices
TrakEM2	Cardona et al.^5^	https://imagej.net/plugins/trakem2/

**Other**		

Confocal laser scanning microscope	Zeiss	LSM800
PELCO BioWave Pro+ microwave processing system	Ted Pella	36700
PELCO SteadyTemp Pro solid state chiller	Ted Pella	50062
PELCO ColdSpot Pro	Ted Pella	36116-10
PELCO EM Pro vacuum chamber	Ted Pella	3536
Aclar film	Agar Scientific	AGL4458
Conductive epoxy glue	ITW Chemtronics	CW2400
Gatan 3View rivet holder	Gatan, Inc.	3VRHBM
Ultramicrotome	Leica Microsystems	EM UC7
Diamond trimming knife	DiATOME	Trim 90
Gatan 3View system SEM pin stubs with large Ø2.4 mm flat, Ø2 mm pin ×12.5 mm H, aluminum	Micro to Nano	10-006002-50
X-ray microscope	Zeiss	XRadia 510 Versa
Sputter coater	Quorum Technologies	Q150R S
3View2XP system	Gatan, Inc.	3View 2XP
Field emission scanning electron microscope	Zeiss	Sigma VP

## Materials and equipment

**Table:** 0.2 M disodium hydrogen phosphate

Reagent	Final concentration	Amount
disodium hydrogen phosphate, anhydrous	0.2 M	14.2 g
ddH_2_O	N/A	N/A
**Total**	**0.2 M**	**500 mL**

Filter sterilize and store at 4°C up to 1 year.

**Table:** 0.2 M sodium dihydrogen phosphate

Reagent	Final concentration	Amount
Sodium dihydrogen phosphate dihydrate	0.2 M	15.6 g
ddH_2_O	N/A	N/A
**Total**	**N/A**	**500 mL**

Filter sterilize and store at 4°C up to 1 year.

**Table:** 0.2 M phosphate buffer (PB)

Reagent	Final concentration	Amount
0.2 M disodium hydrogen phosphate	0.2 M	202.5 mL
0.2 M sodium dihydrogen phosphate	0.2 M	47.5 mL
**Total**	**N/A**	**250 mL**

Store at 4°C up to 3 months.

**Table:** 0.1 M phosphate buffer (PB)

Reagent	Final concentration	Amount
0.2 M disodium hydrogen phosphate	0.2 M	202.5 mL
0.2 M sodium dihydrogen phosphate	0.2 M	47.5 mL
ddH_2_O	N/A	250 mL
**Total**	**N/A**	**500 mL**

Store at 4°C up to 1 month.

**Table:** 4% formaldehyde solution

Reagent	Final concentration	Amount
36% w/v Formaldehyde (EM)	4%	1 mL
0.2 M phosphate buffer	0.1 M	4.5 mL
ddH_2_O	N/A	3.5 mL
**Total**	**N/A**	**9 mL**

Prepare just before use.

**CRITICAL:** Aldehyde fixatives are hazardous. Wear a lab coat and gloves. Must be handled in a fume hood.

**Table:** 4% formaldehyde + 2.5% glutaraldehyde solution

Reagent	Final concentration	Amount
36% w/v Formaldehyde EM	4%	1 mL
25% w/v Glutaraldehyde EM	2.5%	0.9 mL
0.2 M phosphate buffer	0.1 M	4.5 mL
ddH_2_O	N/A	2.6 mL
**Total**	**N/A**	**9 mL**

Prepare just before use.

**CRITICAL:** Aldehyde fixatives are hazardous. Wear a lab coat and gloves. Must be handled in a fume hood.

**Table:** 3% potassium ferricyanide

Reagent	Final concentration	Amount
Potassium ferricyanide (K_3_Fe(CN)_6_)	3%	0.15 g
0.2 M phosphate buffer	0.2 M	5 mL
**Total**	**N/A**	**5 mL**

Store at 4°C up to 1 year.

**Table:** 2% Reduced OsO_4_

Reagent	Final concentration	Amount
Osmium tetroxide 4% Aqueous Solution	3%	2 mL
3% potassium ferricyanide	1.5%	2 mL
**Total**	**N/A**	**4 mL**

Prepare just before use.

**CRITICAL:** Osmium tetroxide is hazardous. Wear a lab coat and gloves. Must be handled in a fume hood.

**Table:** 1% Thiocarbohydrazide (TCH)

Reagent	Final concentration	Amount
Thiocarbohydrazide	1%	0.1 g
ddH_2_O	N/A	10 mL
**Total**	**N/A**	**10 mL**

Prepare before use. Weigh TCH in a glass vial. Add ddH_2_O and warm at 60°C . Swirl the vial every 10 min to facilitate dissolving. Incubate solution at 60°C for more than 1 h until use. Filter through a 0.22 μm Millipore syringe filter right before use.

**CRITICAL:** Solid TCH must be handled in a fume hood.

**Table:** 2% OsO_4_

Reagent	Final concentration	Amount
Osmium tetroxide 4% Aqueous Solution	2%	2 mL
ddH_2_O	N/A	2 mL
**Total**	**N/A**	**4 mL**

Prepare just before use.

**CRITICAL:** Osmium tetroxide is hazardous. Wear a lab coat and gloves. Must be handled in a fume hood.

**Table:** 1% uranyl acetate (UA)

Reagent	Final concentration	Amount
Uranyl acetate	1%	0.5 g
ddH_2_O	N/A	50 mL
**Total**	**N/A**	**50 mL**

Prepare the mixture in a 50-mL conical tube. Place it on a rotator or a shaker until uranyl acetate completely dissolves. Cover completely the tube with aluminum foil to avoid exposure to light. Store at 4°C. Let the tube stand at all times to avoid contamination of UA deposition that may appear at the bottom, and take aliquot from the top. Alternatively, the solution can be filtered just before use using a syringe and a filter with a 0.2 μm pore size. Avoid using the last few milliliters at the bottom of the tube. Discard if the solution becomes cloudy.

**CRITICAL:** Uranyl acetate is hazardous and radioactive. Wear a lab coat and gloves. Solid uranyl acetate must be handled in a fume hood. Care must be taken to avoid contamination of the lab space. Follow necessary regulations for handling, storing and discarding uranyl acetate.

**Table:** 30 mM aspartic acid (pH 3.8)

Reagent	Final concentration	Amount
aspartic acid	1%	0.5 g
ddH_2_O	N/A	50 mL
**Total**	**N/A**	**50 mL**

Dissolve aspartic acid and adjust pH to 3.8 with potassium hydroxide. Filter sterilize and store at 4°C up to 3 months.

**Table:** Walton’s lead aspartate

Reagent	Final concentration	Amount
30 mM aspartic acid (pH 3.8)	30 mM	10 mL
Lead nitrate	20 mM	0.066 g
**Total**	**N/A**	**10 mL**

Prepare just before use. Prepare the mixture in a clean glass vial. Dissolve lead nitrate completely. Adjust pH to 5.5 by adding 1 M potassium hydroxide drop by drop. After each drop, swirl the solution gently to mix and check pH using a pH testing paper. The solution must remain clear. Incubate the solution at 60°C for more than 30 min until use.

**CRITICAL:** Lead nitrate is hazardous. Wear a lab coat and gloves. Must be handled in a fume hood.

**CRITICAL:** Dropwise addition of potassium hydroxide is critical. As pH rises, lead precipitation occurs more easily.

**Table:** 100% dry ethanol

Reagent	Final concentration	Amount
Sodium sulfate anhydrous	N/A	∼150 g
Ethanol, absolute, 99.8+%	N/A	∼300 mL
**Total**	**N/A**	**∼300 mL**

Use approximately 1/5 volume of sodium sulfate to remove water in ethanol. Place sodium sulfate powder on a metal tray. Bake in an oven at 60°C for ≥12 h. Transfer the powder to a 300-mL screw top glass bottle and fill to the brim with ethanol. Shake the bottle, then leave it until the sodium sulfate power settles. Take aliquot from the top of the bottle and keep the bottle always filled with ethanol. Store at 20°C–24°C.

**Table:** Durcupan resin

Reagent	Final concentration	Amount
Durcupan ACM, single component A, M epoxy resin	52.41%	11.4 g
Durcupan ACM, single component B, hardener 964	45.98%	10 g
Durcupan ACM, single component C, accelerator 960 (DY 060)	1.38%	0.3 g
Durcupan ACM, single component D	0.23%	0.05 g
**Total**	**N/A**	**∼20 mL**

Prepare just before use. Place a magnetic stir bar in a disposable plastic beaker. Weigh components A, B, C, and then D. Immediately after adding component D, gently stir the mixture on a stirrer. Check that the color of the mixture became uniform. Keep stirring for at least 10 min. Avoid air bubbles.

**CRITICAL:** Resin components are hazardous. Wear a lab coat and gloves. Must be handled in a fume hood.

## Step-by-step method details

### *In vivo* labeling of cremaster microcirculation, tissue dissection and fixation

**Timing: 6 h**

This section describes *in vivo* fluorescent labeling of cremaster muscle ECs and preparation of the tissue for fixation. *In vivo* labeling is critical for achieving pronounce EC labeling and highlighting the entire vasculature without affecting the ultrastructure.

***Note:*** Use an appropriate number of replicates (ideally tissues should be isolated from 2–3 mice) to make sure finding a ROI close to appropriate landmarks is possible. Preparing replicates is also important in case of sample damage during the preparation.

**Figure 1:**
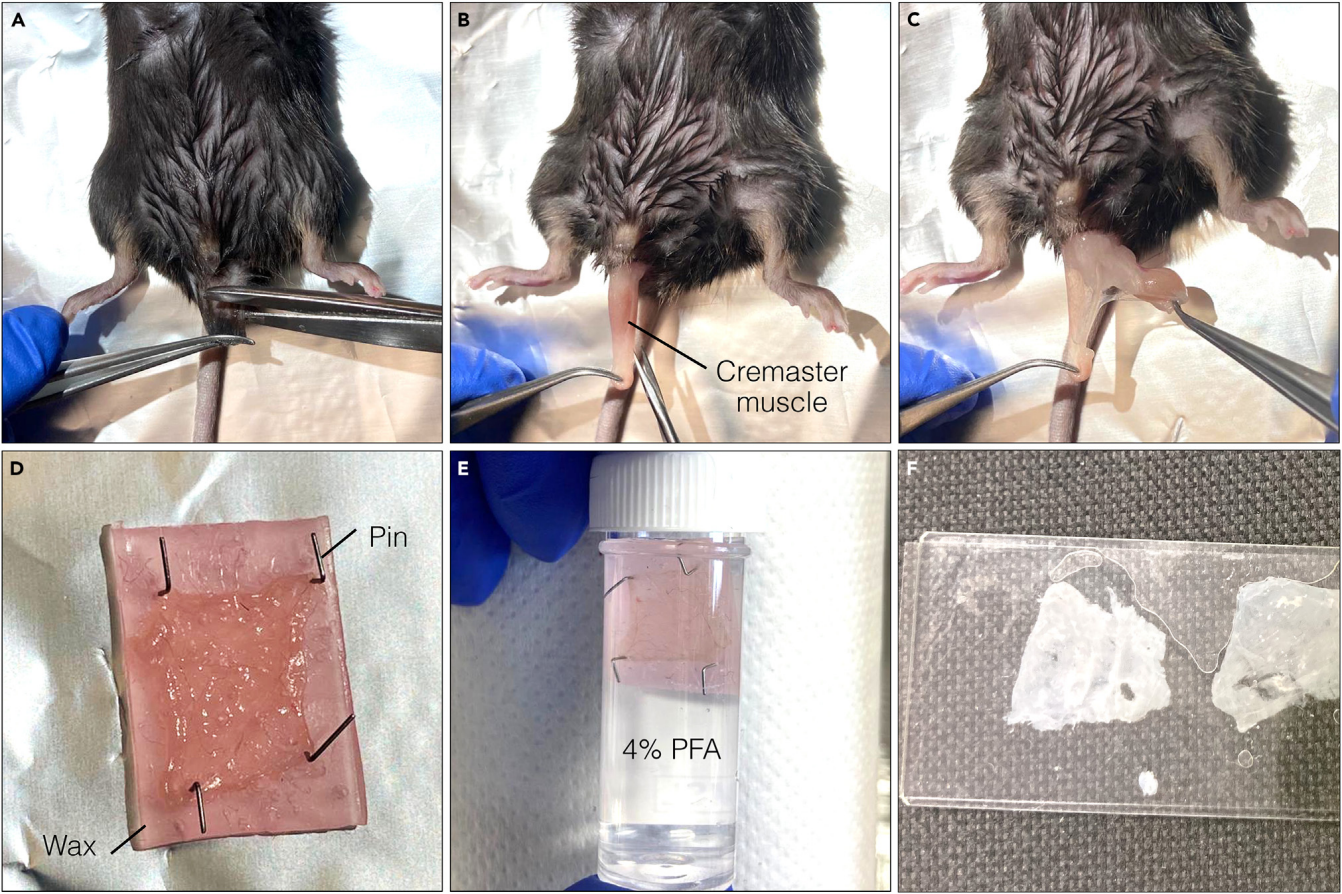
Mouse cremaster muscle tissue dissection and fixation (A) Cut tip of the scrotum. (B) Pull out and cut cremaster muscle. (C) Remove epididymis and testis. (D) Detach cremaster tissue and pin out on dental wax. (E) Fix in 4% formaldehyde solution and (F) mount on a slide.

***Note:*** Treat tissue as gently as possible to minimize morphology artefacts and vessels collapsing.

1. *In vivo* labeling of ECs within cremaster muscle microcirculation.^6^
  ***Note:*** For labeling ECs of the microcirculation, we recommend immunostaining for PECAM-1/CD31. This protein localizes to cell-cell junctions in between ECs and therefore delineates tissue vessels. Immunostaining of other EC junctional markers, such as VE-Cadherin, can also delimit the endothelial layer of the vascular bed.
  a. Conjugate anti-PECAM-1 mAb to the Alexa fluorophore of choice using an antibody labeling kit (see key resources table). Follow manufacturer instructions. For this example, we used Alexa Fluor 488.
  b. Dilute the antibody to a final concentration of 10 μg/mL in a total volume of 400 μL of Dulbecco phosphate buffered saline for the injection.
    ***Note:*** Make sure to prepare the antibody dilution under a laminar flow hood to preserve sterile conditions.
  c. Inject 4 μg of Alexa Fluor 488-labeled anti-PECAM-1 mAb in the intrascrotal cavity of the experimental mouse.
  d. Wait 4 h for appropriate labeling of ECs, before tissue exteriorization and dissection.
2. Cremaster muscle tissue exteriorization and dissection (Figure 1).
  a. Cull mice by using a Schedule 1 humane method (e.g., cervical dislocation).
  b. Cut the tip of the scrotum, allowing exteriorization of the testis.
  c. Make an incision in the cremaster muscle and cut in an anterior direction.
  d. Cut away epididymis and testis and make a proximal cut to detach the cremaster muscle from the animal.
  e. Pin out cremaster muscle on a piece of dental wax to spread the tissue before fixation. Use 4 pins per tissue and cut the piece of dental wax to fit the size of the cremaster muscle tissue.

3. Fix tissue immediately by immersing the pinned-out tissue in 4% formaldehyde solution at 4°C for 40 min. Rinse tissue once with 0.1 M PB with gentle agitation.

### Confocal microscopy

**Timing: 6 h**

For successful ROI targeting in CLXEM, it is important to perform light microscopy at multiple resolutions with effective landmark structures. This section describes the steps to acquire such data without losing positions of ROIs.

**CRITICAL:** Formaldehyde is a relatively weak fixative. To minimize impact on the ultrastructure, perform confocal microscopy as quickly as possible, and fix the tissue with glutaraldehyde (Step 8) immediately after imaging.

4. Mount tissue for confocal fluorescence microscopy as quickly as possible.
  ***Note:*** Ensure that the tissue is submerged in 0.1 M PB at all times to avoid drying.
  a. Spread the tissue on a glass slide.
  b. Carefully cover the tissue with a #1.5 coverslip (24 mm × 50 mm).
  c. Seal the chamber with warm soft wax and let it solidify.
    ***Note:*** Make sure the wax covers the entire coverslip. The seal is critical to prevent leakage and drying of the buffer.
5. Perform confocal imaging using a confocal microscope with a motorized XY stage and ROI saving functionality (Zeiss LSM 800 used in this example).
  a. Calibrate the XY-stage, then load the sample ensuring the slide is pushed to the extreme bottom left and firmly locked between position sliders. This will allow later removal and replacement while retaining ROIs.
  b. Identify a ROI and capture a Z-stack. In this example we acquired a Z-stack at a pixel size of 99 nm and 0.5-μm axial spacing using a Plan-Apochromat 63×/1.40 oil immersion lens. Alexa Fluor 488 and tdTomato were sequentially excited using 488 nm and 561 nm lasers (both at 10% output power in our system), respectively. Fluorescence of Alexa Fluor 488 and tdTomato were collected at 515–565 nm with detector gain 660 V, and at 575–640 nm with detector gain 650 V, respectively. Images were acquired with 4-line averaging.
  c. Store the XYZ position coordinates (under the stage module).

**Figure 2:**
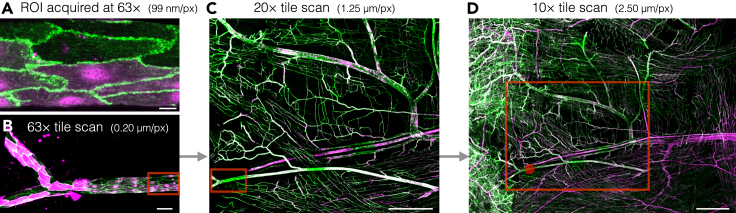
Confocal microscopy of cremaster muscle microcirculation of *Atg5*^*ΔEC*^ mouse at different magnifications ECs immunostained for PECAM-1 (green) were imaged by confocal microscopy. (A–D) Maximum projections of confocal Z-stacks. tdTomato fluorescence (magenta) indicates *Atg5*^*−/−*^ ECs. After capturing the ROI at 63×, tile scans at 63× with FOV from panel A indicated by the red box (B), 20× with FOV from panel B indicated by the red box (C), and 10× with FOV from panel C indicated by the red box (D) were acquired. Red dot in D indicates the position of the ROI. Scale bars: (A) 10 μm; (B) 50 μm; (C) 500 μm; (D) 1000 μm.

***Note:*** In this specific example, we selected a ROI that contained neighboring tdTomato- and tdTomato+ (*Atg5*^*−/−*^) ECs. Since autophagy deficiency in postcapillary venular ECs led to the accumulation of junctional adhesion molecules at cell borders,^1^ we targeted a ROI exhibiting thickened PECAM-1 at EC junctions between tdTomato+ (*Atg5*^*−/−*^) ECs (Figure 2A). Of note, venules can be distinguished from capillaries by diameter (postcapillary venules are between 25–40 μm in diameter).

**CRITICAL:** It is recommended to image the superficial region of the tissue for optimal light penetration and acquisition of maximal fluorescence signal intensity. We acquired a ROI within the first ∼150 μm of the cremaster muscle tissue. This will provide more accurate imaging of vessels and EC morphology.

**CRITICAL:** Make sure the ROI is selected close to a venule with an identifiable shape. We chose a ROI on a “Y” shaped venule (Figure 2B) with a branching point positioned at ∼400 μm from cells of interest, a distance that could fit within the maximum size of the final trimmed resin block, determined by the size limits of the diamond knife and field of view (FOV) in serial block-face scanning electron microscopy (SBF-SEM).

***Note:*** To avoid bleaching of the fluorophores, make sure to acquire high-resolution images of the ROI before the tile scans at lower magnifications (Step 6).

6. Acquire tile scans of the tissue at different magnifications. Start at 63× (Figure 2B), then lower the magnification to 20× (Zeiss Plan-Apochromat 20×/0.8) and 10× (Zeiss EC Plan-Neofluar 10×/0.30) (Figures 2C and 2D).
  a. Acquire tile scans around the ROI(s) at 63×.
  b. Remove immersion oil from the coverslip before switching from an oil to an air lens.
    i. Remove the slide by loosening the right slider only.
    ii. Clean away the oil using lens tissue and 70% ethanol.
  c. Replace the slide in the same orientation by pushing to the extreme bottom left and securing the right slider firmly. This ensures positional data to remain accurate.
  d. Acquire Z-stacked tile scans at 20× and 10×. Make sure to include the ROI(s) and prominent venules and/or vessels as landmark structures.
  e. Create maximum projections of the tile scans using Zeiss ZEN or any desired program. For example, in Fiji, the command can be found under the Image/Stacks/Z project.
  f. Draw the position(s) of the ROI(s) using an annotation tool.
  g. Save the files as annotated copies to use as vasculature maps in the later steps.

**CRITICAL:** Include in the tile scan a few thicker vessels nearby. Tile scans taken at lower magnifications become vasculature maps for later targeting of the ROI.

**Figure 3:**
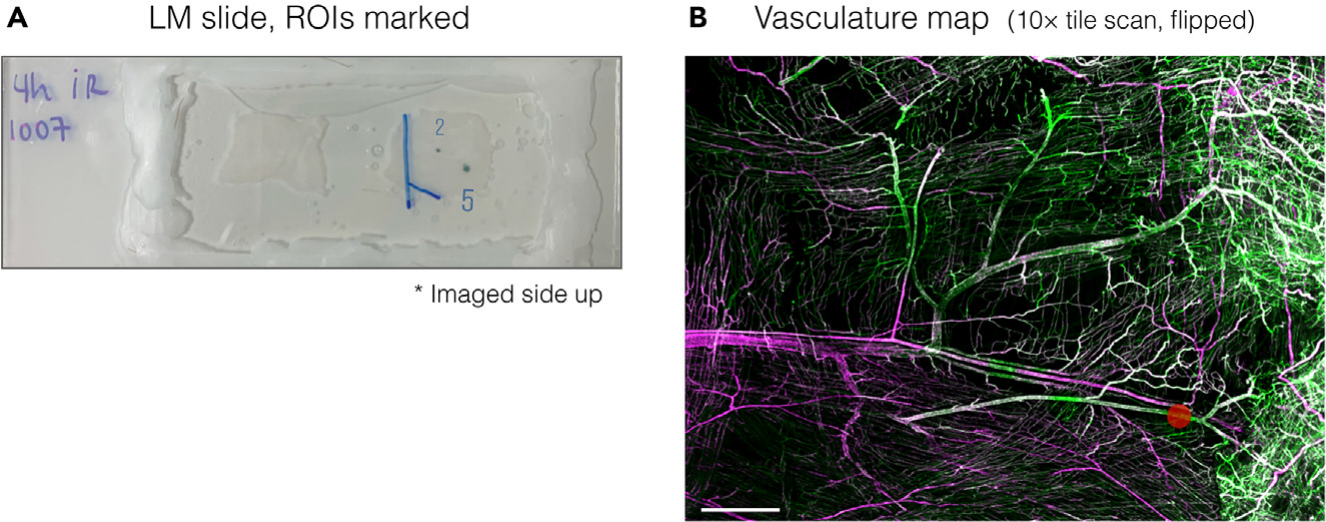
ROI position marking (A) A sample tissue in a LM chamber photographed after confocal microscopy. Positions of two ROIs (“2” and “5”) are marked on the coverslip side (Step 7b). The two blue lines indicate where the tissue was cut after removing from the chamber to make the shape asymmetrical (Step 8b). (B) Position of ROI “5” is indicated with a red dot on the vasculature map. Scale bar, 1 mm.

***Note:*** Any camera can be used as long as the tissue shape and the marks are clearly visible. The tissue shape and the marks become a guide when identifying the ROI before the first X-ray scan.

7. Mark ROI positions onto the slide.
  a. Before removing the slide from the microscope stage, roughly mark the position(s) of the ROI(s) on the backside of the slide with a marker pen by looking straight down at the objective lens.
  b. Remove the slide from the microscope stage.
  c. Copy the marks onto the coverslip.
  d. Take a photo of the entire tissue with the marks (Figure 3A).

**CRITICAL:** When identifying the ROI position on the vasculature map, note which side of the tissue is facing up.

8. Remove the tissue from the slide and fix with formaldehyde and glutaraldehyde.
  **CRITICAL:** The tissue must be fixed with glutaraldehyde immediately after light microscopy. This is critical for ultrastructure preservation.
  a. Carefully remove paraffin seal and float off the tissue in a bath filled with 0.1 M PB.
  b. Trim the tissue with a razor blade, if necessary, to obtain an asymmetrical shape to aid in orientation and tracking of the ROIs in later steps (Figure 3A). Do not cut off too much tissue to avoid causing damage to the vasculature organization.
  c. Immerse the tissue in 4% formaldehyde + 2.5% glutaraldehyde solution for 1 h at 20°C–24°C.
    **Pause point:** Glutaraldehyde fixed tissues can be stored in a formaldehyde solution diluted to 1% with 0.1 M PB at 4°C.
    ***Note:*** To avoid shrinkage of the tissue, we recommend storage in 1% formaldehyde instead of extending the fixation time. In addition, it is recommended to process the tissue for EM as soon as possible to avoid damage.

### Sample preparation for SBF-SEM

**Timing: ∼5 days**

Aldehyde-fixed tissue is post-fixed and stained for SBF-SEM using the National Center for Microscopy and Imaging Research (NCMIR) protocol.^7^ To facilitate this step, we used a previously reported microwave-assisted protocol.^8^ Microwave irradiation facilitates penetration of heavy metals and speeds up the preparation. In the protocol described below, microwave was used only for fixation and staining (Steps 11–25). However, dehydration and resin infiltration can also be successfully performed using microwave.^8^ Although it takes longer (∼6 days in total), sample preparation can be performed without microwave.

***Note:*** All microwave assisted steps are performed using the PELCO SteadyTemp Pro Solid State Chiller and ColdSpot Pro to maintain sample temperature. In addition, the PELCO EM Pro Vacuum Chamber was used to aid penetration of solutions into the tissue. The step-by-step microwave program is provided as supplementary material.

**CRITICAL:** All steps below should be performed in a fume hood including the microwave-assisted steps.

9. Prepare all the solutions required for Steps 10–25.

***Note:*** Some solutions must be prepared just before use as indicated in the materials and equipment section. If pausing experiment after Step 13, plan and prepare solutions accordingly.

***Note:*** In Steps 10–25, use ≥2 mL of solutions including the rising steps.

10. Rinse aldehyde-fixed tissue.
  a. Transfer tissue to a plastic petri dish of convenient size filled with 0.1 M PB (in our case, a 35-mm dish was used).
  b. Rinse the tissue with ∼2 mL of 0.1 M PB 3 times for 5 min each.
11. Fix the tissue with reduced OsO_4_.
  a. Exchange 0.1 M PB with 2% reduced OsO_4_.
  b. Transfer the dish with the lid closed to the microwave (PELCO BioWave Pro+ Microwave Processing System) using the vacuum chamber.
  c. Remove the lid of the petri dish and incubate the sample with microwave on/off cycles for 14 min (see Steps 1–7 of the microwave program in Table S1).
12. Rinse the tissue in the fume hood.
  a. Rinse once with 0.1 M PB.
  b. Rinse with ddH_2_O until the solution becomes clear.
13. Wash twice with ddH_2_O in the microwave (see Steps 10–11 of the microwave program in Table S1).

**CRITICAL:** Rinse thoroughly to reduce residual OsO_4_ as it reacts with TCH.

**Pause point:** Osmicated tissue can be stored in ddH_2_O at 4°C, however, it is recommended to complete the preparation as soon as possible to avoid damage.

14. Treat the tissue with TCH.
  a. Transfer 1% TCH to a syringe with a 0.22 μm Millipore syringe filter attached.
  b. Remove ddH_2_O from the tissue and add filtered TCH.
  c. Transfer the dish with the lid closed to the microwave device in a vacuum chamber.
  d. Remove the lid from the petri dish and incubate the sample with microwave on/off cycles for 14 min (see Steps 12–18 of the microwave program in Table S1).

***Note:*** Program is set to heat samples to 40°C.

15. Rinse the tissue in the fume hood with ddH_2_O until the solution becomes clear.
16. Wash twice with ddH_2_O in the microwave device (see Steps 20–21 of the microwave program in Table S1).

***Note:*** Residual TCH could remain as a grayish film at the water surface. Use a fresh petri dish for the next step.

17. Treat the tissue with OsO_4_.
  a. Prepare 2% OsO_4_ in a fresh petri dish and transfer the tissue.
  b. Place the dish with the lid closed into the microwave in the vacuum chamber.
  c. Remove the lid from the petri dish and incubate the sample with microwave on/off cycles for 14 min (see Steps 22–28 of the microwave program in Table S1).
18. Rinse the tissue with ddH_2_O until the solution becomes clear.
19. Wash twice with ddH_2_O in the microwave (see Steps 30–31 of the microwave program in Table S1).
20. Treat the tissue with uranyl acetate.
  a. Exchange ddH_2_O with 1% uranyl acetate.
  b. Transfer the dish with the lid closed to the microwave in the vacuum chamber.
  c. Remove the lid from the petri dish and incubate the sample with microwave on/off cycles for 14 min (see Steps 32–38 of the microwave program in Table S1).

***Note:*** Program is set to heat up samples to 40°C.

21. Rinse the tissue with ddH_2_O until the solution becomes clear.
22. Wash twice with ddH_2_O in the microwave device (see Steps 40–41 of the microwave program in Table S1).
23. Treat the tissue with lead aspartate.
  a. Exchange ddH_2_O with lead aspartate.
  b. Transfer the dish with the lid closed to the microwave in the vacuum chamber.
  c. Remove the lid from the petri dish and incubate the sample with microwave on/off cycles for 14 min (see Steps 42–48 of the microwave program in Table S1).

***Note:*** Program is set to heat up samples to 50°C.

24. Rinse the tissue with ddH_2_O until the solution becomes clear.
25. Wash twice with ddH_2_O in the microwave device (see Steps 50–51 of the microwave program in Table S1).

**Pause point:** Sample can be stored in ddH_2_O at 4°C, however, it is recommended to complete the process as soon as possible to avoid damage.

26. Dehydrate the sample with graded series of ethanol.
  a. Prepare 30, 50, 70 and 90% ethanol in ddH_2_O.
  b. Incubate the sample in each ethanol solution for 30 min each at 20°C–24°C. Place the dish on a laboratory shaker to apply gentle agitation during dehydration.
  c. Incubate in 99.8% ethanol (straight from the bottle) then 100% dry ethanol for 30 min each at 20°C–24°C with gentle agitation.
27. Transfer the sample to a glass vial containing propylene oxide and incubate twice for 15 min each.

**CRITICAL:** Use glass, polypropylene, or aluminum, but not polyethylene containers for propylene oxide.

28. Prepare Durcupan resin as indicated in the materials and equipment section.

***Note:*** Durcupan resin mixture can be prepared during steps 26–27.

29. Infiltrate the sample with 50% Durcupan resin diluted in propylene oxide.
  a. Prepare 1:1 mixture of propylene oxide and Durcupan. Mix until the mixture becomes uniform.
  b. Exchange propylene oxide with the 50% Durcupan.
  c. Incubate the sample 12–24 h in a fume hood or a desiccator. Leave the lid open and allow propylene oxide to gradually evaporate.
30. Infiltrate the sample with fresh 100% Durcupan resin.
  a. Prepare fresh Durcupan resin as indicated in the materials and equipment section.
  b. Transfer the sample to an aluminum dish with fresh resin.
  c. Briefly place the dish on a hot plate to decrease viscosity of the resin to mix residual old resin into the fresh resin.
  d. Infiltrate 2 h at 20°C–24°C.

***Note:*** The sample can be placed on a platform rocker or a rotator (if the container using with lid) to aid infiltration if available.

**Figure 4:**
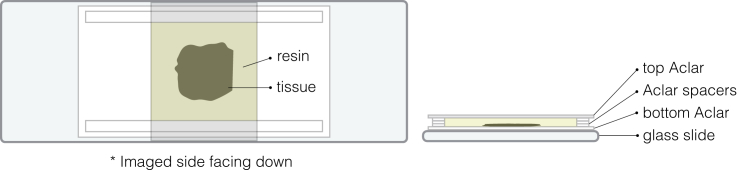
Flat embedding of resin-infiltrated tissue Top (left) and side (right) views of a flat-embedding chamber. Top Aclar film is shown in gray.
  b. Prepare several thin strips of Aclar film as spacers.
  c. Place the bottom Aclar sheet and spacers on a glass slide. Adjust the height of the chamber using the spacers to fit the tissue.
  d. Then place the infiltrated tissue with the imaged side facing down. Add some fresh resin, then slowly cover the tissue with the top Aclar sheet as illustrated in Figure 4. Remove any excess resin.

31. Exchange resin with fresh 100% Durcupan resin.
  a. Transfer the sample to a new aluminum dish with fresh Durcupan.
  b. Briefly place the dish on a hot plate to decrease viscosity of the resin to mix residual old resin into the fresh resin.
32. Flat embed the tissue.
  a. Prepare two sheets of Aclar film. Cut the top sheet to size so that it is just big enough to cover the tissue (Figure 4).

**CRITICAL:** As the superficial venules of the tissue were imaged by confocal microscopy, the side facing the objective lens is oriented facing downwards in the flat-embedding chamber in order to place the ROI closer to the block face.

**CRITICAL:** Do not press the top Aclar sheet. Make sure to fill enough resin in the chamber and to check there are no bubbles trapped between the tissue and the top Aclar sheet.

33. Polymerize resin in an oven at 60°C for 48 h.
34. Remove the sample from the oven. Carefully peel away the Aclar films.

### First trimming (X-ray scan 1, trimming 1)

**Timing: ∼3 days**

This section describes the first step of the sequential trimming and X-ray imaging. The protocol below including second and third X-ray μCT (Steps 39 and 41, respectively) are adjusted to the Zeiss Xradia Versa 510 X-ray microscope, however, any high-resolution X-ray μCT system can be used.

**Figure 5:**
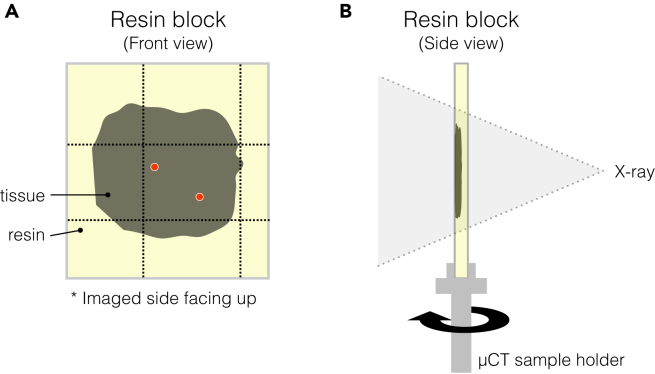
Preparation for X-ray scan 1 (A) Front view of the flat embedded tissue in the resin block. Approximate positions of the ROIs are shown in red dots. A rectangle scratched around the ROIs is indicated with dotted lines. (B) Side view of the resin block mounted on a Zeiss Versa sample holder. Directions of X-ray and stage rotation of X-ray scan 1 are indicated.
  b. Mark the resin surface to roughly indicate the region that contains the ROIs.
    i. Identify the region that contains the ROIs using the photo of the tissue and positions of the ROIs (Figures 5A and 6A).

35. Prepare the block for X-ray scan 1.
  a. Place the imaged side of the tissue up. The asymmetry of the tissue shape should indicate the orientation (Figures 3A and 5A).

**Figure 6:**
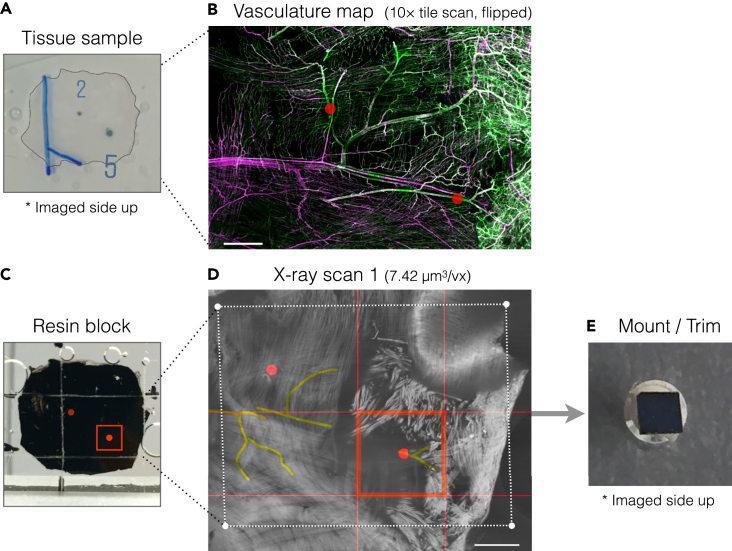
Trimming 1 guided by X-ray scan 1 and vasculature map (A) A sample tissue marked and photographed after confocal microscopy. (B) 10× tile scan of the tissue used as a vasculature map for trimming 1. Positions of the two ROIs (“2” and ”5″) are marked with red dots (B–D). (C) Resin block with a scratched rectangle encompassing the ROIs. (D) A tomographic slice of X-ray scan 1. The area of the scratched rectangle in (C) is shown as a white rectangle. (E) Trimmed block mounted on a Gatan 3View system SEM pin stub (hereinafter referred to as aluminum pin). The trimming area is indicated by red squares (C, D). Scale bars: (B, D) 1 mm.
    ii. Use any pointy tool such as a thin syringe needle or a razor blade and apply scratches to draw a rectangle (or any shape) on the resin block surface (Figures 5A and 6C). The rectangle becomes a guide for Trimming 1.

***Note:*** For the ease of finding the marks in X-ray data, apply thick enough scratches that are easily visible by eye.

**CRITICAL:** When marking on the resin surface, make sure not to damage the block or the tissue.

36. Acquire X-ray scan 1.
  a. Mount the resin block on a Zeiss Versa sample holder with screw clamp (Figure 5B) and place it in an X-ray microscope (Zeiss XRadia 510 Versa).
  b. Set the FOV to include the selected region. If the applied mark is not visible, decide the FOV based on tissue shape.
  c. Check the rotation center of the block. The selected region should be inside the FOV at all rotation angles.
  d. Scan the sample at source voltage 40 kV, power 3 W and exposure time 13 s using a 0.4× objective. Acquire 1601 projections at a voxel size of 7.4 μm^3^. The acquisition takes ∼9 h including warm up.
    ***Note:*** The resolution of the first X-ray scan should be high enough to resolve large blood vessels, which are the first landmarks for Trimming 1. In our system, they were clearly identifiable at a voxel size of ∼7 μm^3^.
  e. Export reconstructed dataset as individual tiff files.
    **CRITICAL:** Make sure the pixel size information is correctly stored in the final tiff file.
    **CRITICAL:** Make sure the side of the tissue imaged by confocal microscopy is facing up.
37. Trim the block (Trimming 1) around the ROI (Figures 6D and 6E).
  a. Find the rectangle mark (applied at Step 35b) in the X-ray scan 1 data.
    i. Open the exported data of X-ray scan 1 in 3dmod of IMOD software^2^ using slicer mode, or any other desired program that allows reslicing of 3D data.
    ii. Reslice data to fit to the block surface plane where the rectangle is marked.
    iii. Go through the slices and find the rectangle and landmark vessels. Adjust the image thickness in 3dmod and show average projection of multiple slices, if necessary, to visualize the rectangle and vessels.
  b. Annotate rectangle corners and landmark vessels (Figure 6D). Use any desired program. In our case, vessels were annotated using a projection of several slices of 3dmod snapshots using Adobe Photoshop (Adobe, San Jose, USA) or Fiji.
  c. Decide the area for Trimming 1.
    i. Measure distances from the corners of the rectangle.
    ii. Mark the block according to the measurements (Figure 6D).
  d. Cut out an area larger than the defined trimming area using a fresh Teflon-coated razor blade.

**CRITICAL:** When trimming, make sure to use a fresh Teflon-coated razor blade. Slow and stepwise cutting into resin is recommended to avoid cracks and damage.

**CRITICAL:** Make sure not to trim away prominent landmark vessels at this stage.

**CRITICAL:** Cut off one corner of the block in order not to lose the orientation.

38. Mount the block onto an aluminum pin.
  a. Mix the two components of the conductive epoxy glue (ITW Chemtronics).
  b. Apply to an aluminum pin and place the sample block onto the glue.
  c. Roughly adjust the block face to horizontal position.
    i. Before the epoxy resin sets, insert the pin to a Gatan 3View Rivet Holder and mount it to an ultramicrotome.
    ii. Set segment arc tilt at 0°.
    iii. Place a diamond trimming knife at rotation 0°.
    iv. Match roughly the block face (around the ROI) to the cutting plane of an ultramicrotome. Carefully move the orientation of the block on the pin by pushing the block corners using a fine tweezer.
      **CRITICAL:** Retract knife when touching the block on the aluminum pin.
      ***Note:*** Mounting the block face horizontally brings the cutting plane of SBF-SEM closer to the imaging plane of confocal microscopy.
      ***Note:*** It is not mandatory to match the block face to the cutting plane as the required orientation of the sectioning depends on the sample. We recommend checking the X-ray scan 1 dataset to roughly decide the sectioning orientation before mounting the block on the pin.
  d. Polymerize the conductive resin at 60°C for ≥12 h.
  e. Complete Trimming 1 using a fresh Teflon-coated razor blade (Figure 6E).

### Second trimming (X-ray scan 2, trimming 2)

**Timing: 2 days**

This section describes the second step of the sequential trimming and X-ray imaging to obtain the final block for SBF-SEM.

39. Acquire X-ray scan 2.
  a. Insert the aluminum pin with the mounted sample into the Zeiss Versa sample holder with a pin vice.
  b. Set the FOV to include the entire block. Check the rotation center of the block. All four corners of the block face should be inside the FOV at all rotation angles.
  c. Scan the sample at source voltage 40 kV, power 3 W and exposure time 5 s using a 4× objective. Acquire 1601 projections at a voxel size of 3.26 μm^3^. The acquisition takes ∼4.5 h including warm up.
  d. Export the reconstructed dataset as individual tiff files.

***Note:*** The resolution of the second X-ray scan should be high enough to resolve the target venule. In our setup, it was clearly identifiable at a voxel size of 3.26 μm^3^.

**Figure 7:**
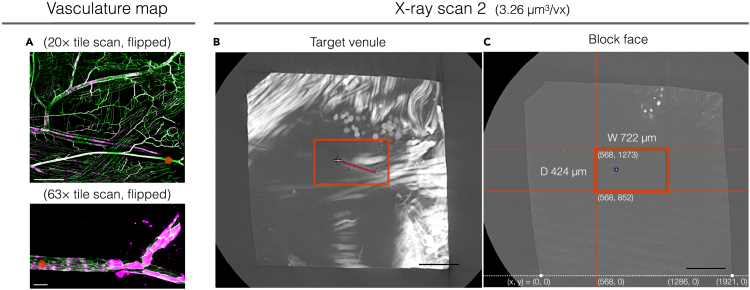
Annotations for Trimming 2 guided by vasculature maps and X-ray scan 2 (A) Vasculature maps with position of the ROI indicated by red dots. (B) A slice parallel to the block face plane in the X-ray scan 2 data showing the target venule. White cross indicates the position of the ROI identified in the X-ray scan 2 based on the distance of the ROI from the branch point of the Y-shaped venule. The area for the final block face is shown by a red rectangle. (C) Block face plane in the X-ray scan 2 data. The area for the final block face is shown by a red rectangle in the center. The positions for trimming 2 were determined by measuring distances from block corners as indicated. Scale bars: (A) 500 μm (top) and 50 μm (bottom); (B, C) 400 μm.
  c. Create 3dmod snapshots of slices at the block face and at the plane containing the ROI together with the annotation (Figures 7B and 7C).
  d. In the snapshot of the block face plane, measure the position of the final block face using block corners.
  e. At an ultramicrotome, mount the sample and place a 90° trimming diamond knife at a knife angle 0°.
  f. Orient the block at an angle required for the trimming and align the block face to the cutting plane.
  g. At the leading edge of the block face, bring the knife edge of the 90° trimming diamond knife to the position measured in Step 40d using the measuring mode of the Leica UC7 ultramicrotome.
  h. Trim the final block.
    ***Note:*** It is recommended to use a 90° trimming diamond knife to keep the size of the block face uniform during the SBF-SEM.

40. Trim around the ROI (Trimming 2).
  a. Find the landmark vessels in X-ray scan 2 data.
    i. Open the data of X-ray scan 2 using 3dmod slicer mode (or any other desired program).
    ii. Reslice the data to fit to the block face plane. Show average projection of multiple slices if necessary.
    iii. Go through the slices and find the landmark vessels.
  b. View the slice containing the target venule at zoom factor 1.0 in 3dmod slicer mode. Select and mark the area of the final block face for Trimming 2 using the 3dmod “rubber band” tool (red rectangles in Figure 7).
    **CRITICAL:** It is important to include within the final block the landmark which was imaged together with the ROI by confocal microscopy (Steps 5, 6; Figure 2B). In our case the final block face was selected to include the Y-shaped branch of the target venule.
    ***Note:*** We adjusted the block size to fit within the FOV of the Zeiss Versa 510 X-ray microscope at a resolution of ∼1 μm^3^/voxel. This size also fits the range of knife movement in our Gatan 3View2XP system, which is 1200 μm.

### ROI targeting (X-ray scan 3)

**Timing: ∼2 days**

In this section, the positions of the target cells are identified within the final resin block for SBF-SEM by correlating high-resolution X-ray μCT of the final resin block and the confocal microscopy data.

41. Acquire X-ray scan 3.
  a. Insert the aluminum pin with the mounted sample into the Zeiss Versa sample holder with a pin vice and set up the scan as described in Step 39.
  b. Scan the sample at source voltage 40 kV, power 3 W and exposure time 20 s using 4× objective. Acquire 1601 projections at a voxel size of 1.03 μm^3^. The acquisition takes ∼11.5 h including warm up.
    ***Note:*** The resolution of the final X-ray scan should be high enough to resolve nuclei. In our setup, they were clearly identifiable at a voxel size of ∼1.0 μm^3^.
    ***Note:*** All structures visible in the final X-ray data may become useful landmarks for later steps such as depth estimation of the ROI and SBF-SEM FOV adjustments. Set up the final X-ray scan at a maximum resolution possible according to the size of the final block face. We recommend ∼1.0 μm^3^/voxel or below.
  c. Export the reconstructed dataset as individual tiff files.
  d. Open the data of X-ray scan 3 using 3dmod slicer mode (or any other desired program). Reslice data to fit to the block face plane. Show average projection of multiple slices if necessary.
  e. Create 3dmod snapshots of the volume that contains the block face and the tissue.
42. Correlate the 63× tile scan and the resliced X-ray scan 3.
  a. Open the 63× tile scan data and the X-ray scan 3 data in Fiji.
    ***Note:*** X-ray data can be downscaled, if necessary, if the landmarks (nuclei) are clearly discernible. Additionally, it could be resliced parallel to the block face plane if necessary.
  b. Open the Fiji plugin BigWarp,^4^ and choose the 63× tile scan data as the “moving image” and X-ray scan 3 data as the “target image” (Figures 8A and 8B).
    ***Note:*** For details of the BigWarp plugin, please refer to the plugin page in the ImageJ Docs.

**Figure 8:**
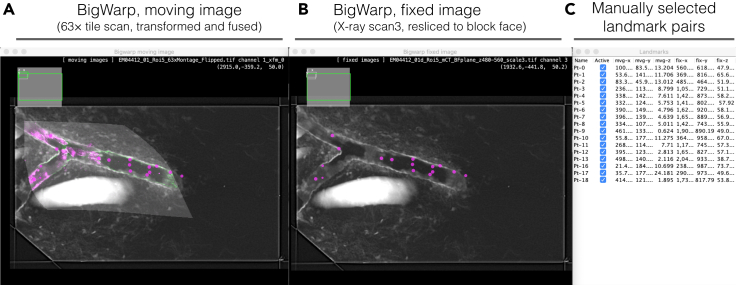
Correlation of light and X-ray data using BigWarp Moving image (fluorescence data) (A) is transformed based on the manually selected 18 landmark pairs (C) and fused with the fixed image (B). See also Video S1.
  c. Go through the volumes and manually choose landmark pairs in the moving and target images. Specify positions of the pairs as accurately as possible. We used the outline of the venule and nuclei of endothelial cells as landmarks (Figure 8).
    ***Note:*** Try transforming the moving image using different transformation types available in the plugin. Adding more landmarks usually helps to get better result. Transformation and fusing also helps identifying more landmark pairs. It is recommended to select landmarks not only around the target cells but in the peripheral regions for better results.
  d. Once achieving successful transformation of the moving image, export the warped moving image and create a multichannel stack in Fiji to overlay on the target image (Video S1).
    Video S1. Overlay of correlated light and X-ray data sets created at step 42 Transformed confocal 63× tile scan data overlaid on X-ray scan 3 data. Scale bar, 100 μm.
    Video S2. X-ray scan 3 data used for FOV determination at SBF-SEM (step 43, 46) X-ray scan 3 data resliced to fit the block face plane and rotated to match the orientation of the sample block in the SBF-SEM. Region to acquire (FOV) is shown as a red box. The positions of nuclei of the target cells are annotated with pink dots. Scale bar, 100 μm.
  e. Export the landmarks as csv file. The command can be found in the landmark window under File/Export landmarks.
    ***Note:*** The exported landmark csv file can be imported to retrieve the landmarks in the BigWarp, if the same pair of tiff files are used as moving and target images.
43. Find the cells of interest captured in the 63× confocal data using the warped and overlaid image. Annotate their nuclei in the X-ray scan 3 data for further guidance at the SBF-SEM (Figure 9; Video S2).

**Figure 9:**
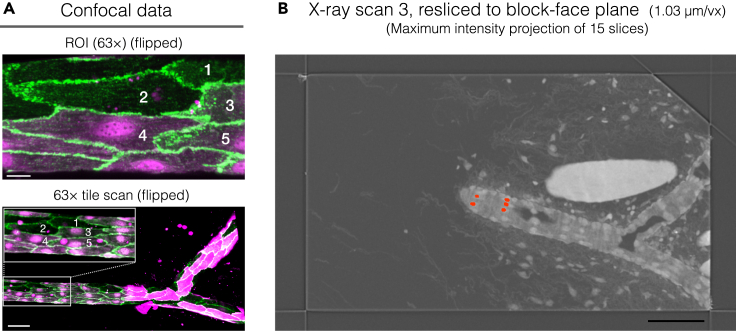
Identification of target cells in X-ray scan 3 Positions of the target cells are annotated with numbers (1–5) in the confocal data (A) of 63× acquisition (top) and 63× tile scan (bottom), and their nuclei are marked red in the X-ray scan 3 (B). Maximum projection of 15 slices (∼15 μm) is shown for better visualization of the target cell nuclei. See also Video S2. Scale bars: (A) 10 μm (top) and 50 μm (bottom); (B) 100 μm.

### Serial block-face SEM

**Timing: ≥2 days**

In this section, SBF-SEM of the target cells is performed using the X-ray μCT data of the final resin block as a guide. Position and depth of the target cells are determined from the annotated X-ray scan 3 data created in the previous section.

44. Coat the block with 10-nm of platinum with a sputter coater (Quorum Technologies, Q150R S) to increase conductivity of the block.

***Note:*** Other sputtering target such as gold or gold-palladium can be used to coat the block.

**Figure 10:**
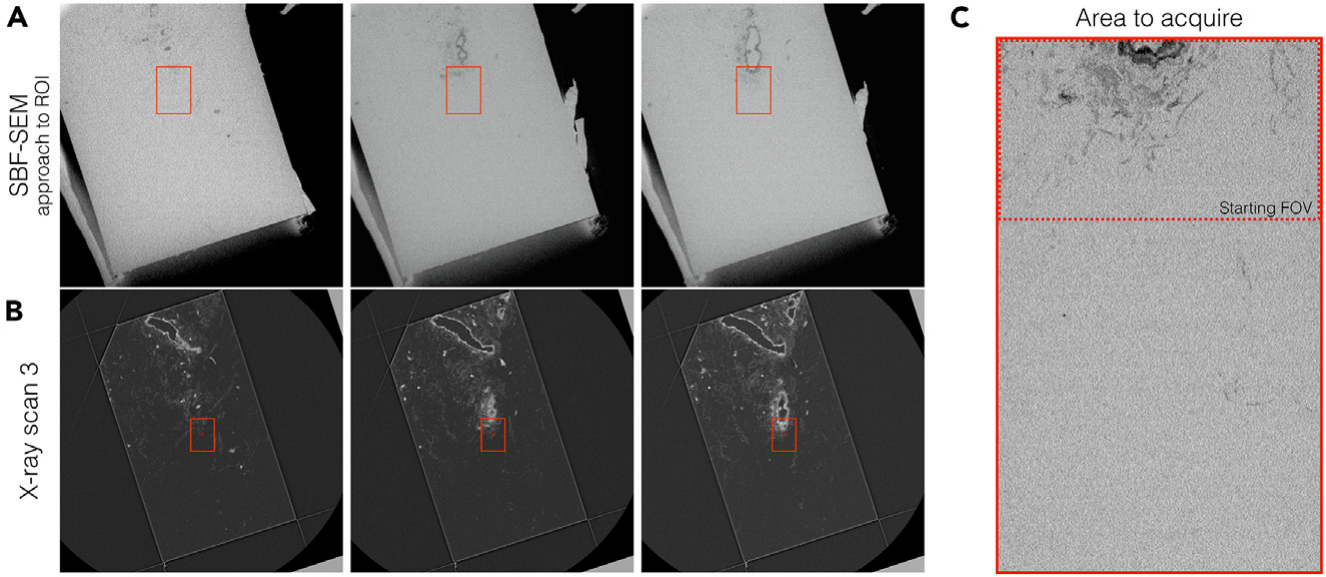
Approaching ROI for SBF-SEM (A) Inverted backscattered electron images acquired during the approach cutting to the ROI. Target venule is appearing at the block face. (B) Corresponding slices in the X-ray scan 3 data. FOV is shown as a red box (Step 46). (C) The entire area on the block face to acquire SBF-SEM data. The FOV at the start of the run is indicated as a dotted red rectangle.

***Note:*** If necessary, modify the slicing angle of the X-ray scan 3 to fit the actual cutting plane. The X-ray data becomes a guide for the FOV set up.

45. Set the sample in the SBF-SEM chamber.
  a. Place the sample in the chamber of a SBF-SEM (Sigma VP, Zeiss; 3View2XP, Gatan Inc.) and position the block at the center of the motion range of the 3View knife.
  b. Set the sample stage at “stroke up” position (the level the sectioning occurs). Elevate the sample holder until the diamond knife edge reflection on the block face becomes thin. Fasten the sample holder.
  c. Start the cutting cycle at 200-nm thickness and continue until the knife touches the block face.
  d. Place the needle of the Focal Charge Compensator^9^ above the block face,^10^ and close the chamber door and pump the chamber.
46. Decide the imaging FOV for SBF-SEM in the X-ray scan 3 data (Video S2).
  a. Open the annotated X-ray scan 3 data (created in Step 43) in 3dmod slicer mode and reslice to fit the block face.
  b. Decide and select the area of the SBF-SEM FOV and place a mark using the 3dmod “rubber band” tool (red rectangle in Video S2).
  c. Estimate the depth of the ROI from the block face.
47. Start the cutting cycle at 200-nm thickness to approach the ROI. Acquire low magnification back-scattered electron images of the block face and monitor sectioning using the X-ray scan 3 data (Figure 10).

48. Once the cutting plane nears the ROI, set the FOV for high resolution acquisition. In our sample, as there was some angle between the block face and the longitudinal axis of the target venule, we set the starting FOV as indicated in Figure 10C (red dotted rectangle) and laterally shifted it towards the bottom as sectioning proceeded.

**CRITICAL:** When shifting the FOV, make sure to keep enough overlap in order not to miss any volume (see Video S3).

49. Set the SBF-SEM imaging parameters and start the run.

***Note:*** Our acquisition parameters are as follows: Accelerating voltage 1.8 keV; aperture 30-μm, dwell time 2.0 μs; slice thickness 50 nm; lateral resolution 8.44 nm/pixel.

***Note:*** The nitrogen gas output of the FCC is controlled by its percentage output level. This should be optimized for each system and sample to get the best balance between imaging and cutting. Adjust the percentage value according to the level of sample charging. We recommend below 80% as higher gas flow could interfere with the detector.

Video S3. Correlated light and electron microscopy data sets created at step 51 Warped 63× confocal data overlaid to SBF-SEM data (scaled down to 200 nm/px). Note that shifting of the FOV during the acquisition run caused loss of a portion of the volume below the ROI.

### 3D correlation of LM and EM datasets

**Timing: 2–3 days**

In this last section, the SBF-SEM data is correlated with the 63× confocal data to identify the target cells in the EM data. This allows selection of the regions for further analysis.

**Figure 11:**
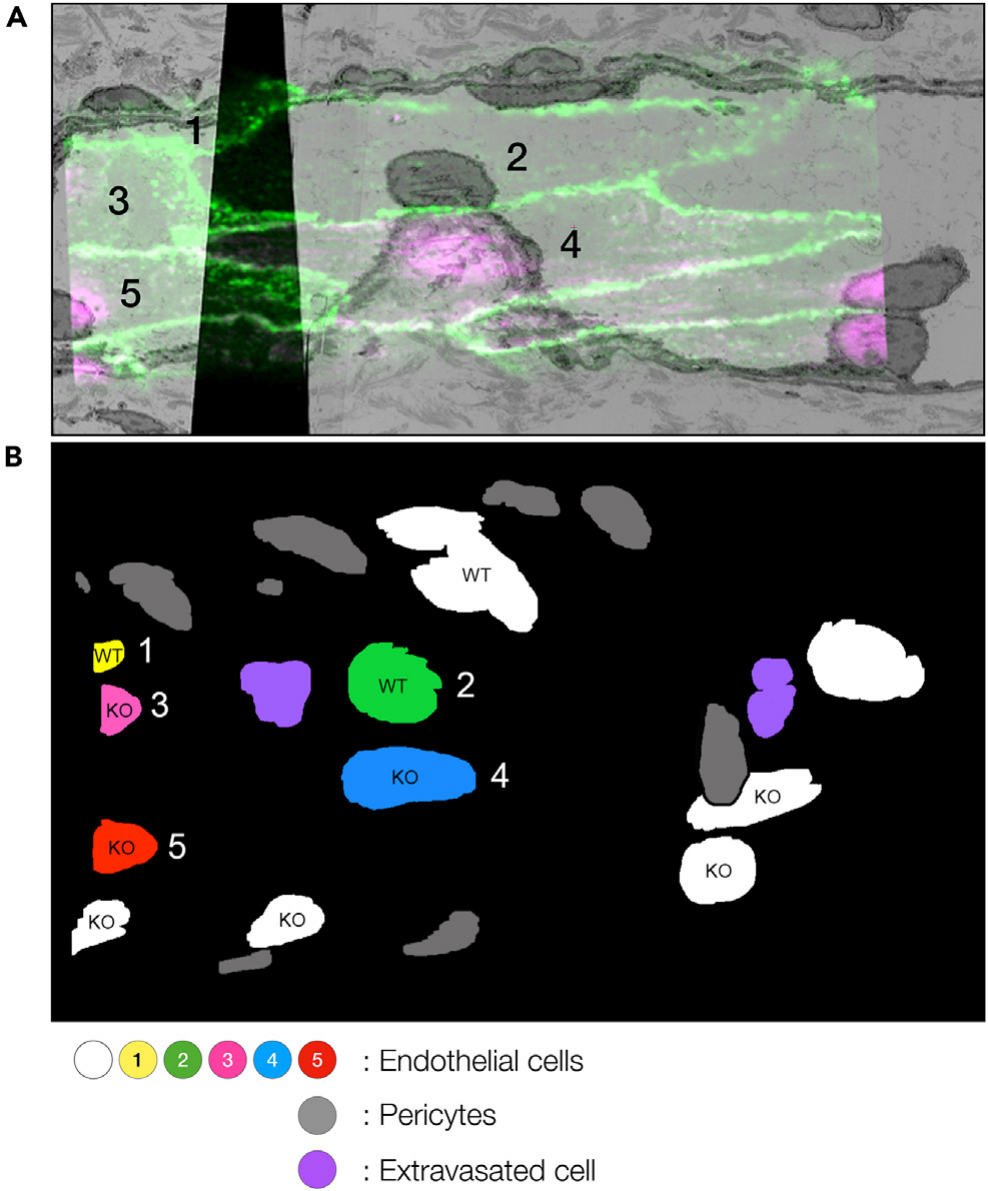
Nuclei map annotated with cell number and cell types (A) A snapshot of the CLEM data (Video S3). (B) Maximum intensity projection of nuclei segmentation.

***Note:*** Once the target cells are identified, more “area lists” can be added to the same TrakEM2 xml file if necessary for other annotations and segmentations of structures of interest.

50. Align the SBF-SEM dataset.
  a. Convert the raw SBF-SEM data to tiff files and create a stack.
  b. Align the stack using the Fiji plugin Register Virtual Stack Slices using desired image transformation models.
    ***Note:*** We recommend translation for the image transformation model to start with.
    ***Note:*** Step 50c–e can be skipped if no further alignment correction is required after Step 50b.
  c. Start the Fiji plugin TrakEM2,^5^ import the stack aligned in Step 50b, and save the TrakEM2 xml file.
  d. Use the “Align layers manually with landmarks” function of the TrakEM2 to further correct the alignment.
  e. Export the aligned dataset as a tiff stack.
    ***Note:*** “Align layers manually with landmarks” function of the TrakEM2 is useful when aligning between stacks with different FOVs. For details of the Fiji plugins, please refer to the websites listed in the key resources table.
    ***Note:*** As the purpose of the correlation is to identify target cells in the EM volume, the EM dataset can be scaled down before the next step if necessary.
51. Overlay the 63× confocal data to the aligned SBF-SEM data using BigWarp.
  a. Open both the 63× confocal data and the aligned SBF-SEM stack in Fiji.
  b. Start BigWarp and choose the 63× confocal data as the “moving image” and the aligned SBF-SEM stack as the “target image”.
  c. Carefully go through the volumes and manually choose landmark pairs in the moving and target images. We used the outline and nuclei of endothelial cells as landmarks.
  d. Once successful transformation of the moving image has been achieved, export the warped moving image and create a multichannel stack in Fiji to overlay on the target image (Video S3).
  e. Export the landmarks as csv file as described in Step 42e.
52. Create a nuclei map to identify positions of target cells and other cell types in the region around the ROI.
  a. Open the TrakEM2 xml file of the aligned SBF-SEM stack created at Step 50c.
  b. Create “area lists” to quickly annotate nuclei of endothelial cells and other cells.
  c. Export “area lists” and combine maximum projections to obtain a nuclei map (Figure 11B). Annotating positions of all target cells and cell types helps to extract information of the tissue from the CLEM data.

## Expected outcomes

**Figure 12:**
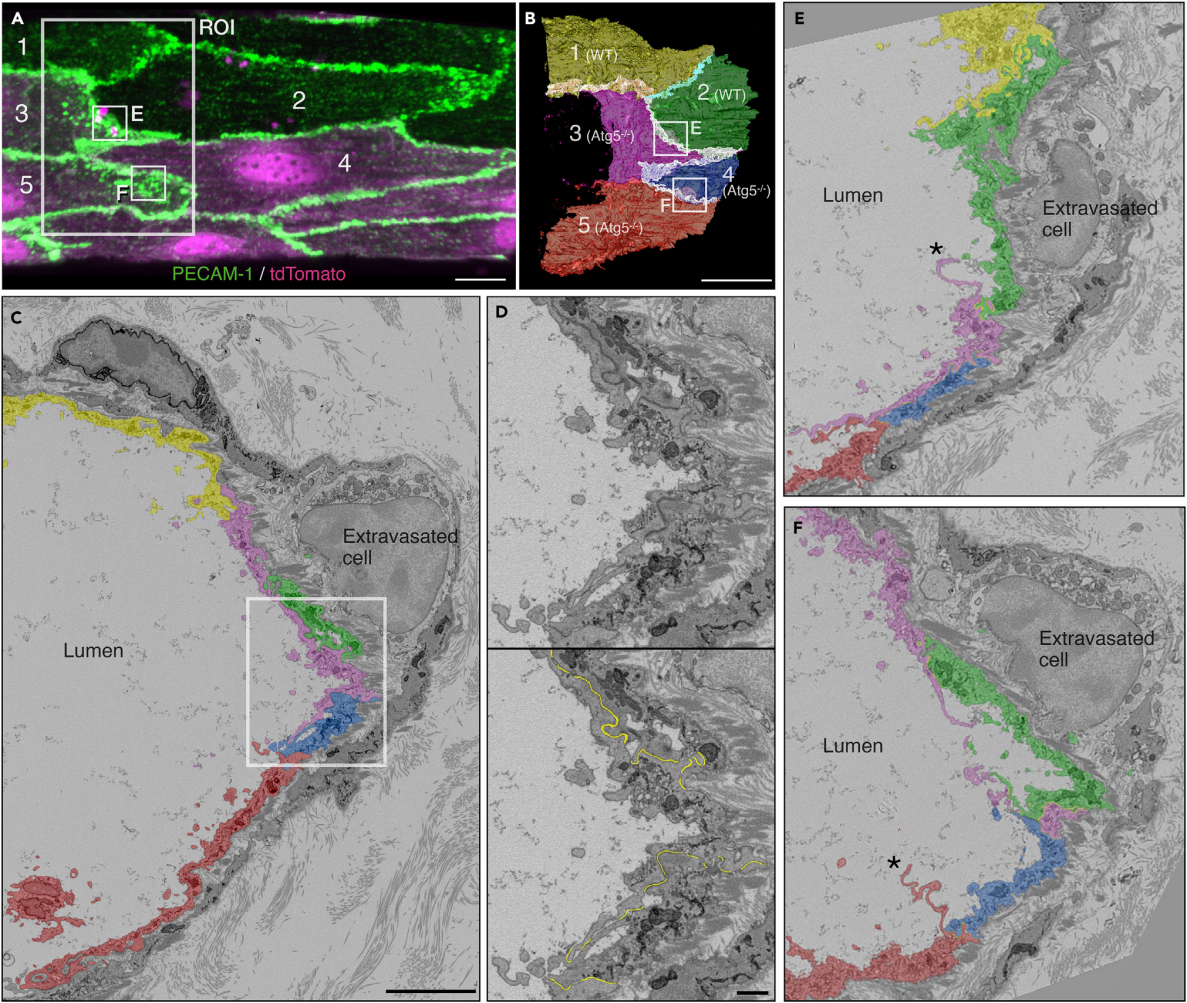
Cell-cell contacts and membrane shape of target endothelial cells (A) 63× confocal microscopy image of the cell-cell contact region that involves five WT and Atg5^−/−^ endothelial cells. The five cells (1–5) in the ROI are indicated with numbers. (B) 3D model of the plasma membrane of the five cells indicated in (A). All the cell-cell contact regions are overlaid and shown in light blue (WT-WT contact) or white (WT-Atg5^−/−^ and Atg5^−/−^-Atg5^−/−^) lines. Apposing plasma membranes with spacing less than or equal to 25 nm were segmented and modeled as contact regions.^1^ (C) An SBF-SEM slice from the ROI. (D) Enlarged view of the region indicated with white box in (C). Cell-cell contact regions observed as electron dense lines (top) were segmented as shown in yellow (bottom). (E and F) Sheet-like plasma membrane projections of Atg5^−/−^ cells that extend towards the venule lumen were found at the cell-cell borders with expanded PECAM-1 staining indicated with boxes in (A) and (B). An extravasated cell which was expected at the expanded PECAM-1 region as a response to inflammatory stimuli was also found in the same region. Scale bars: 5 μm (A–C), 1 μm (D).

Accurate ROI targeting facilitates data acquisition in volume CLEM. The targeting workflow presented here is based on the vasculature pattern and nuclei that are both visible in light and X-ray microscopy. As these structures can be fluorescently labeled without damaging ultrastructure, they have been used successfully in CLXEM workflows.^11^^,^^12^ Precise targeting also minimizes the FOV to maximize the resolution of SBF-SEM image acquisition. In our sample, this allowed accurate detection of PECAM-1-dependent cell-cell contacts between ECs (Figure 12D) based on distances between plasma membranes of two neighboring ECs.^1^ Targeted vEM of contacts between *Atg5*^*−/−*^ ECs identified by light microscopy revealed PECAM-1 positive enlarged contact sites and abnormal membrane shapes (“membrane flaps or projections”) as compared to contacts between wild type (WT) ECs (Figures 12B, 12E and 12F). Such information was key to understanding the role of autophagy in the modulation of EC junctions during inflammation.^1^

Nuclei annotation within the targeted volume created a useful map to grasp the positions of WT and *Atg5*^*−/−*^ ECs together with the surrounding other cell types that are not visible in the fluorescence data (Figures 11 and 12). The map was also useful to prioritize the laborious manual segmentation of cell-cell borders.

We described here a targeting workflow for SBF-SEM of thin translucent tissue samples visualized by confocal laser scanning microscopy. As the imaging depth of μCT exceeds that of light microscopy, the protocol is applicable to light microscopy techniques with increased imaging depth such as wide field and multiphoton microscopy. Likewise, any downstream vEM modality, such as array tomography, focused ion beam SEM, or TEM could be used.

## Limitations

The CLXEM workflow described here relies on chemical fixation and pre-embedding light microscopy. Relatively weak fixation by formaldehyde and/or prolonged absence of fixative during light microscopy could lead to alterations in the morphology of sensitive structures. One should be cautious of the effects of chemical fixation on structures of interest. To preserve unstable structures, it is possible to combine low concentration (e.g., 0.05%–0.5%) of glutaraldehyde with 4% formaldehyde. However, because glutaraldehyde induces a much stronger autofluorescence than formaldehyde, the optimum concentration is greatly affected by the fluorescence intensity of the structures of interest. One should carefully adjust and optimize the concentration.

When using confocal laser scanning microscopy, the ROI needs to be positioned relatively close to the tissue surface for optimal light penetration and fluorescence signal detection. The working distance of a high numerical aperture objective lens will also be a limitation (working distance of a typical 63x/1.4 objective lens is ∼0.2 mm). For these reasons, in the current protocol, the confocal microscopy was limited to superficial vessels. For samples that require fluorescence imaging deeper into the specimen, improved light penetration can be achieved by combining other fluorescence microscopy techniques, such as multiphoton microscopy, into the workflow. However, for high-resolution imaging, the working distance of high numerical aperture objective lenses may remain the limiting factor, demanding a trade-off between imaging depth and resolution. Light-sheet microscopy can also be combined if the sample preparation required for imaging does not impair ultrastructure. Tissue clearing methods which requires lipid extraction are often not suitable for CLEM. However, recently developed aqueous clearing methods of brain tissues are reported to be compatible with EM.^13^^,^^14^

Shrinkage and warping during dehydration can cause problems with the overlay of the LM and EM data. This should not be a critical problem when using structures with the size of nuclei for registration. However, it becomes more problematic as the target ROI becomes smaller. In such cases, incorporating additional landmarks of smaller sizes and higher density needs to be considered.

High resolution X-ray μCT systems can be expensive. If there is not a system readily available in a nearby life sciences imaging facility, consider approaching physical or material sciences facilities, or apply for hard X-ray imaging beam time at a synchrotron. In the UK, access to laboratory-based X-ray CT can be gained through the National X-ray Computed Tomography (NXCT).

## Troubleshooting

### Problem 1

Orientation of the tissue is lost during EM sample preparation.

### Potential solution

If the tissue orientation is lost after marking the ROI on the coverslip during EM sample preparation (Steps 8–32), flat embed the tissue as normally but try using minimum amount of resin to position the ROI close to the resin block surface. Compare the tissue shape with the photo taken at Step 7b and find the orientation. If the orientation is still not clear, X-ray μCT of the wider area of the tissue could be acquired before or instead of the X-ray scan 1 (Step 36). Compare the data with the low magnification tile scans (Step 6) to find the orientation. However, the resolution of this X-ray scan should be high enough to identify the landmark structures (thick vessels and venules in our sample) captured in the tile scans.

To avoid the problem, make sure to prepare the tissue in an asymmetric shape. Taking photos of the tissue at multiple steps of the sample preparation also helps to keep track of the orientation. Dissection microscope with a digital camera can be useful for this. Preparing replicates is important as it becomes more difficult to rescue the sample safely if it is mounted up-side-down in conductive resin on an aluminum pin.

### Problem 2

Tissue broke or deformed during EM sample preparation.

### Potential solution

During fixation, staining and dehydration (Steps 11–27), some tissues might become more brittle and break apart, and some might curl and lose its original shape. If the behavior of the tissue during EM sample preparation is unknown, prepare a test sample and perform a test preparation. Attaching tissue to a glass coverslip coated with positively charged substances such as poly-L-lysine, or with tissue adhesives such as CELL-TAK (Corning 354240, 354241) may help avoiding tissue deformation.

Alternatively, 1.5%–2% low melting point agarose can be used to mount the tissue on a substrate. In the case of agarose, residual fixative in the agarose layer could react with the following fixative and causes unwanted staining of the agarose layer. Make sure to minimize the thickness of the agarose layer. Optimize the rinsing step during fixation and staining (Steps 11–25). In addition, the temperature of lead aspartate treatment (Step 23) should be decreased to 40°C to avoid agarose melting. The staining property does not change at 40°C in our hands^15^; however, the protocol should be optimized for each sample. For the details of SBF-SEM sample preparation of samples on glass substrate, please refer to Russell et al.^16^

### Problem 3

Resin block cracked during trimming.

### Potential solution

If the resin block got cracks which affect the area that contains the ROI(s), put the pieces together and re-embed in the same resin. Flat embed using the same chamber (Figure 4). In the X-ray scan 1 data, judge if the sample can be still used for targeting the ROI(s).

### Problem 4

Suboptimal density of landmarks for light and X-ray dataset correlation (step 42).

### Potential solution

Nuclei are convenient landmarks for correlation, however, in the mouse model used in this example, the density of tdTomato positive cells in the confocal dataset could be lower than optimum depending on the efficiency of Cre-recombination. Therefore, as described by Bushong et al.,^12^ other nuclear stains such as DRAQ5, DAPI or Hoechst could be introduced prior to confocal microscopy to label nuclei of all cells including tdTomato-negative ECs.

### Problem 5

Suboptimal density of landmarks for light and electron dataset correlation (step 51).

### Potential solution

When correlating two datasets, assigning landmark pairs both within and outside the ROI increases accuracy. For successful correlation, acquire a SBF-SEM volume larger than that of the 63x confocal microscopy. The 63x tile scan (acquired at step 5) can be used for the light and electron microscopy dataset correlation.

## Resource availability

### Lead contact

Further information and requests for resources and reagents should be directed to and will be fulfilled by the lead contact, Azumi Yoshimura (azumi-y@yamaguchi-u.ac.jp).

### Technical contact

Questions about the technical specifics of performing the protocol should be directed to and will be answered by the technical contact, Lucy Collinson (Lucy.Collinson@crick.ac.uk) and Azumi Yoshimura (azumi-y@yamaguchi-u.ac.jp).

### Materials availability

This study did not generate new unique reagents.

### Data and code availability

Original data have been deposited to BioStudies: S-BSST1339 (https://doi.org/10.6019/S-BSST1339) and the Electron Microscopy Public Image Archive (EMPIAR): EMPIAR-11958.

## Acknowledgments

This work was principally funded by the Wellcome Trust (098291/Z/12/Z, to S.N.). N.R.-R. is a Ramón y Cajal awardee (RYC2021-031221-I) funded by the Spanish Ministry of Innovation and Science (MCIN) and Spanish State Research Agency (AEI), cofunded by “NextGenerationEU,” and additionally supported by “Generación de Conocimiento” projects programme of MCIN/AEI (PID2022-137552OA-100). The work in the lab of L.C. was supported by The Francis Crick Institute, which receives its core funding from Cancer Research UK (CC1076), the UK Medical Research Council (CC1076), and the Wellcome Trust (CC1076).

## Author contributions

Conceptualization, N.R.-R., S.N., L.C., and A.Y.; methodology, investigation, and validation, N.R.-R., L.P.-G., R.S.S., S.N., L.C., and A.Y.; writing – original draft, N.R.-R. and A.Y.; writing – review and editing, all authors; funding acquisition, S.N. and L.C.; supervision, L.C.

## Declaration of interests

The authors declare no competing interests.

## Supplemental information

Table S1. Microwave program for PELCO BioWave® Pro+ microwave processing system, related to step 11–25
