# Supplementary material for: Protocol for volume correlative light X-ray and electron microscopy of endothelial cells in mouse tissue
Source: STAR Protoc. 2024 Sep 1;5(3):103257. doi: 10.1016/j.xpro.2024.103257 (PMC11419916; doi:10.1016/j.xpro.2024.103257)
Supplement: Table S1. Microwave program for PELCO BioWave® Pro+ microwave processing system, related to step 11–25 [file mmc1.pdf]

Table S1. Microwave program for PELCO BioWave® Pro+ Microwave Processing System.

Step #	Description	Time (min)	Time (sec)	Power (W)	SteadyTemp temp (ºC)	Vacuum cycle Vent time:	Vacuum cycle Vacuum time:	Vacuum set point:	User Prompt 1 = ON, 0 = OFF	Vacuum OFF	Vacuum AUTO (cycle)	Vacuum ON
1	Osmium ON	2	0	100	21	0	0	20	1	0	0	1
2	Osmium OFF	2	0	0	21	0	0	20	0	0	0	1
3	Osmium ON	2	0	100	21	0	0	20	0	0	0	1
4	Osmium OFF	2	0	0	21	0	0	20	0	0	0	1
5	Osmium ON	2	0	100	21	0	0	20	0	0	0	1
6	Osmium OFF	2	0	0	21	0	0	20	0	0	0	1
7	Osmium ON	2	0	100	21	0	0	20	0	0	0	1
8	BENCH STEP Rinse in PB	0	0	0	21	0	0	0	1	1	0	0
9	BENCH STEP Rinse in water	0	0	0	21	0	0	0	1	1	0	0
10	Rinse in water	0	40	250	21	0	0	0	1	1	0	0
11	Rinse in water	0	40	250	21	0	0	0	1	1	0	0
12	TCH ON	2	0	100	40	0	0	20	1	0	0	1
13	TCH OFF	2	0	0	40	0	0	20	0	0	0	1
14	TCH ON	2	0	100	40	0	0	20	0	0	0	1
15	TCH OFF	2	0	0	40	0	0	20	0	0	0	1
16	TCH ON	2	0	100	40	0	0	20	0	0	0	1
17	TCH OFF	2	0	0	40	0	0	20	0	0	0	1
18	TCH ON	2	0	100	40	0	0	20	0	0	0	1
19	BENCH STEP Rinse in water	0	0	0	21	0	0	0	1	1	0	0
20	Rinse in water	0	40	250	21	0	0	0	1	1	0	0
21	Rinse in water	0	40	250	21	0	0	0	1	1	0	0
22	Osmium ON	2	0	100	21	0	0	20	1	0	0	1
23	Osmium OFF	2	0	0	21	0	0	20	0	0	0	1
24	Osmium ON	2	0	100	21	0	0	20	0	0	0	1
25	Osmium OFF	2	0	0	21	0	0	20	0	0	0	1
26	Osmium ON	2	0	100	21	0	0	20	0	0	0	1
27	Osmium OFF	2	0	0	21	0	0	20	0	0	0	1
28	Osmium ON	2	0	100	21	0	0	20	0	0	0	1
29	BENCH STEP Rinse in water	0	0	0	21	0	0	0	1	1	0	0
30	Rinse in water	0	40	250	21	0	0	0	1	1	0	0
31	Rinse in water	0	40	250	21	0	0	0	1	1	0	0
32	Uranyl acetate ON	2	0	100	40	0	0	20	1	0	0	1
33	Uranyl acetate OFF	2	0	0	40	0	0	20	0	0	0	1
34	Uranyl acetate ON	2	0	100	40	0	0	20	0	0	0	1
35	Uranyl acetate OFF	2	0	0	40	0	0	20	0	0	0	1
36	Uranlyl acetate ON	2	0	100	40	0	0	20	0	0	0	1
37	Uranyl acetate OFF	2	0	0	40	0	0	20	0	0	0	1
38	Uranyl acetate ON	2	0	100	40	0	0	20	0	0	0	1
39	BENCH STEP Rinse in water	0	0	0	40	0	0	0	1	1	0	0
40	Rinse in water	0	45	250	40	0	0	0	1	1	0	0
41	Rinse in water	0	45	250	40	0	0	0	1	1	0	0
42	Lead aspartate ON	2	0	100	50	0	0	20	1	0	0	1
43	Lead aspartate OFF	2	0	0	50	0	0	20	0	0	0	1
44	Lead aspartate ON	2	0	100	50	0	0	20	0	0	0	1
45	Lead aspartate OFF	2	0	0	50	0	0	20	0	0	0	1
46	Lead aspartate ON	2	0	100	50	0	0	20	0	0	0	1
47	Lead aspartate OFF	2	0	0	50	0	0	20	0	0	0	1
48	Lead aspartate ON	2	0	100	50	0	0	20	0	0	0	1
49	BENCH STEP Rinse in water	0	0	0	40	0	0	0	1	1	0	0
50	Rinse in water	0	45	250	40	0	0	0	1	1	0	0
51	Rinse in water	0	45	250	40	0	0	0	1	1	0	0
